# Verbal Encouragement and Between-Day Reliability During High-Intensity Functional Strength and Endurance Performance Testing

**DOI:** 10.3389/fphys.2019.00460

**Published:** 2019-04-25

**Authors:** Florian A. Engel, Oliver Faude, Sarah Kölling, Michael Kellmann, Lars Donath

**Affiliations:** ^1^Department of Movement and Training Science, Institute of Sport and Sport Science, Heidelberg University, Heidelberg, Germany; ^2^Department of Sport, Exercise and Health, University of Basel, Basel, Switzerland; ^3^Unit of Sport Psychology, Faculty of Sports Science, Ruhr University Bochum, Bochum, Germany; ^4^Department of Sport Science, Stellenbosch University, Stellenbosch, South Africa; ^5^School of Human Movement and Nutrition Sciences, The University of Queensland, St. Lucia, QLD, Australia; ^6^Department of Intervention Research in Exercise Training, German Sport University Cologne, Cologne, Germany

**Keywords:** verbal encouragement, functional training, high intensity power training, crossover, performance, RCT

## Abstract

As verbal encouragement (VE) is used in high intensity functional exercise testing, this randomized controlled crossover study aimed at investigating whether VE affects high intensity functional strength and endurance performance testing. We further examined between-day variability of high intensity functional strength and endurance performance testing with and without VE. Nineteen experienced athletes (seven females and 12 males, age: 23.7 ± 4.3 years) performed a standardized one repetition maximum (1 RM) squat test and a 12-min high-intensity functional training (HIFT) workout [as many repetitions as possible (AMRAP)] on four different days over a 2-week period. Athletes randomly performed each test twice, either with VE or without (CON), with a minimum of 72 h rest between tests. Very good to excellent relative between-day reliability with slightly better values for strength testing (ICC: 0.99; CV: 3.5–4.1%) compared to endurance testing (ICC 0.87–0.95; CV: 3.9–7.3%) were observed. Interestingly, VE led to higher reliability during endurance testing. Mean squat strength depicted higher strength values with VE (107 ± 33 kg) compared to CON (105 ± 33 kg; *p* = 0.009, Cohen’s d: 0.06). AMRAP in the endurance test showed negligible differences between VE (182 ± 33 AMRAP) and CON (181 ± 35 AMRAP; *p* = 0.71, Cohen’s d: 0.03). In conclusion, the effects of VE do not notably exceed day-to-day variability during high intensity functional strength (CV: 3.5–4.1%) and endurance (CV: 3.9–7.3%) testing. However, high intensity functional strength and endurance testing with VE seems to be slightly more reliable, particularly during endurance testing.

## Introduction

High-intensity functional training (HIFT) comprises functional power and functional strength elements in addition to high intensity endurance exercises (Glassman, 2007). Contrary to traditional high-intensity interval training, rest periods during HIFT are kept to a minimum. Sustained power output and increased work capacity per time are intended (Glassman, 2007; Sprey et al., 2016). HIFT workouts employ a variety of functional movements executed at varying intensity levels for varying time durations (Smith et al., 2013; Fisker et al., 2016). Since 2007, an annual competition in HIFT with a worldwide open qualifier system evolved to a professional competition and challenges the world’s fittest athletes. Strength exercises such as one repetition maximum (1 RM) trials during squatting, deadlifting, or Olympic lifting on the one hand, and endurance workouts performed for fastest time or “as many rounds/repetitions (reps) as possible” (AMRAP) on the other hand, are performed in training and competition (Smith et al., 2013).

Since most HIFT workouts are performed in groups with a strong and competitive community setting it can be observed that coaches, colleagues, or spectators commonly intensively apply verbal encouragement (VE). Therefore, this training atmosphere makes HIFT interesting for research purposes about the effects of VE on strength and endurance performance (Partridge et al., 2014). Besides assessing the impact of VE on HIFT-specific strength and endurance performance, reliable assessment of physical performance (e.g., strength and endurance) under such conditions with low between-day variability is needed in order to successfully detect cross-sectional or longitudinal changes of physical performance (Atkinson and Nevill, 1998). Therefore, potential effects of VE need to be reflected toward between-day reliability of strength and endurance testing (Faude et al., 2012; Donath and Wolf, 2015). Since recent research demonstrated that during maximal exercise testing VE caused considerable variations in endurance performance (McCormick et al., 2015; Midgley et al., 2018) and force production (Anzak et al., 2011; Belkhiria et al., 2018), a careful evaluation of the effects of VE during HIFT specific exercise testing seems to be necessary.

A supposed underlying mechanism for improved performance in exercise testing while application of VE is the startle mechanism (Carlsen et al., 2007; Anzak et al., 2011), which represents a defensive reflex in the brain stem as a response to loud acoustic stimuli (Gruner, 1989). Additional causes for improved performance with VE are assumed in increased arousal (Giray and Ulrich, 1993) and greater maximal efforts (Andreacci et al., 2002; Anzak et al., 2011). A recent study by Belkhiria et al. (2018) suggests that VE leads to an increased neuromuscular activation during isometric handgrip contractions in untrained individuals. The authors assume that VE increases the specific neural drive, including increases in motor unit recruitment and firing frequency.

To the best of our knowledge, no cross-sectional crossover study on the association between VE and between-day reliability has been conducted in HIFT settings. Since HIFT is a fast growing and worldwide practiced exercise mode (Claudino et al., 2018), which evokes simultaneously relevance of HIFT in the sports and exercise science community, the assessment of HIFT-specific exercise tests for reliability and between-day variability seems to be warranted in order to accurately detect cross-sectional or longitudinal changes of HIFT-specific performance. Therefore, the aim of this study was (a) to evaluate the effects of VE on performance during a HIFT-specific strength (STR) and endurance workout (ENDU) (Andreacci et al., 2002; Binboğa et al., 2013; Obmiñski and Mroczkowska, 2015) and (b) to estimate the changes of absolute and relative between-day reliability measures.

## Materials and Methods

### Participants and Study Design

Nineteen healthy and experienced HIFT athletes, seven female (age 23.7 ± 4.0 years) and 12 male (age 23.7 ± 5.4 years) were enrolled in this randomized controlled cross-over study. Participants can be regarded as experienced athletes as they had practiced HIFT for at least 1 year with a training frequency of at least three times per week for the last 6 months (Table 1). The participants were active at a Gym at the time of recruitment, received relevant study information, including the potential risks and benefits and signed a written informed consent prior to the start of the study.

**Table 1:** Demographic information of the participants.

		Males (*n* = 12)	Females (*n* = 7)
Age (years)		23.7 ± 5.4	23.7 ± 4.0
Training experience (years)	<1	1	0
	≥1	3	4
	2-3	7	3
	>3	1	0
Training days (per week)	1–3	1	3
	>3	8	3
	>5	2	1
	>6	1	0
Training volume (hours/week)	1–3	1	2
	>3	6	2
	>5	3	2
	>7	2	1
Current 1 RM squat (kg)		128.6 ± 23.1	73.9 ± 16.0


*Notes: 1 RM, one repetition maximum*.

**FIGURE 1:**
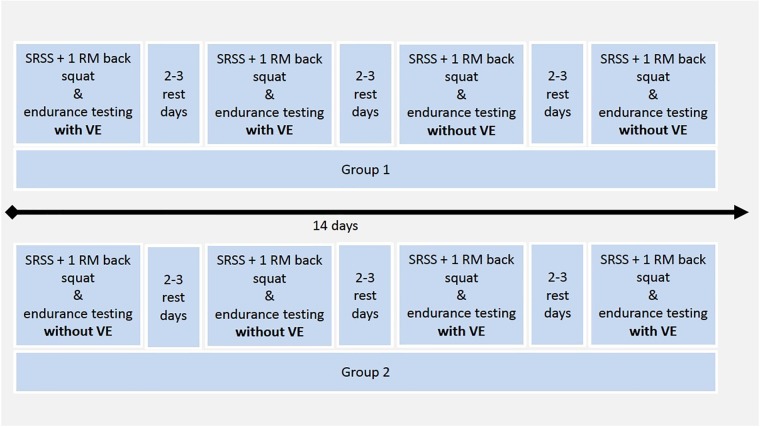
Visualization of the block randomized controlled cross-over design during 14 days. *Notes*: 1 RM, one repetition maximum. SRSS, Short Recovery and Stress Scale. VE, verbal encouragement.

The study fulfilled the criteria of the Code of Ethics for human experimentation, the Declaration of Helsinki (World Medical Association, 2013) and was approved by a Swiss ethics committee *Ethikkommission Nordwest- und Zentralschweiz* (EKNZ^1^ approval number: 2016-1950). All athletes underwent standardized HIFT-specific strength (1 RM back squat) and endurance testing (specified workout of the day) on four different days with a minimum rest period of 72 h in between testing days within 2 weeks. Participants performed tests in a block-randomized order (Figure 1). The randomization procedure involved the following procedures: participants were randomized to group 1 and group 2. Subsequently, the assignment of the order of conditions (VE or control condition) was randomized to the groups. This randomization resulted in group 1 performing the VE condition on day 1 and day 2 and group 2 performing the control condition on day 1 and day 2 (see Figure 1). The testing procedure was performed at a local HIFT Gym where the participants trained regularly. Habitual physical activity was maintained and a training diary was kept during the entire period. Athletes were asked not to engage in strenuous exercise 24 h before each trial.

### Testing Procedure

Prior to the start of each testing session participants filled out the Short Recovery and Stress Scale (SRSS, Kellmann et al., 2016; Kellmann and Kölling, 2019). Subsequently, athletes performed an individual and standardized 10-min warm-up followed by the 1 RM test with the back squat to determine the heaviest weight in kg. Immediately following the 1 RM test, athletes performed the 12-min functional high intensity endurance test, scoring the total repetitions accomplished. Standardized VE was applied by the researcher on interventional-setting days. No VE was applied during the control condition (CON) days. For details of each test and the VE condition, see sections below.

### Strength Testing

After an individual and standardized 10-min warm-up (movement flow consisting of bodyweight squat variations, lunges, and hip mobility drills), athletes performed as many warm-up sets and 1 RM attempts in the back squat as they needed (but not exceeding five to eight sets with increasing weights) to successfully squat their heaviest weight possible on that day within a 20-min time frame. The 1 RM was performed with an Olympic sized 20 kg barbell for the men and a 15 kg barbell for the women. Assessment of 1 RM started with three repetitions at 60% of estimated or current 1 RM, one repetition at 80% of estimated or current 1 RM, and one repetition at estimated or current 90% 1 RM, followed by 1 RM attempt. Subsequently, athletes were allowed to perform additional 1 RM attempts within 20 min, respectively, five to eight sets with increasing weights. Following each successful 1 RM attempt, barbell weight was increased in consultation with the athlete by 1.0–5.0 kg. The 1 RM attempts were terminated if athletes were not able to lift or the lifting-technique was not adequate to HIFT movement standard. The accomplished heaviest weight in kg was considered as strength (STR). The squat attempt was valid if the movement started and ended with the barbell on the back, hips, and knees fully extended and reaching adequate depth during the squat with the hip crease below the top of the knee in the bottom position. This range of motion for squatting movements counts as an official movement standard in HIFT training and competition (CrossFit Inc, 2017).

### Endurance Testing

Within 12 min, the participants tried to complete AMRAP of the following exercises in the given order: 6 burpees over the barbell, 8 barbell ground to overhead lifts, and rowing 250 m on a rowing ergometer. All movements during the endurance testing were performed according to HIFT-standard range of motion (CrossFit Inc, 2017). For the movement “barbell ground to overhead lift,” an Olympic sized 20 kg barbell for the men and a 15 kg barbell for the women was used and loaded with bumper weight plates of standard Olympic diameter for a total weight of 42.5 kg for the men and 30 kg for the women (weight of the barbell included). The chosen weight on the barbell is frequently used in classic HIFT training workouts and competition events and athletes are used to handling these weights for higher repetitions in conditioning settings. The rowing distance was completed on a Concept II indoor rower (PM5). Every completed 10 m on the rower counted as one repetition (as it is generally handled in HIFT competitions) to not outweigh the effort of performing the other exercises where every completed repetition counted as one repetition with a total of 39 repetitions for one completed round (CrossFit Inc, 2017). Number of total repetitions accomplished was considered as endurance (ENDU).

### Verbal Encouragement

No VE was given by the instructor on non-encouragement days (CON). On VE days, positive VE was given before and during 1 RM attempts by the researcher. VE was applied in a 60 s encouragement interval during the 12 min of the endurance test, as frequent VE is assumed to lead to greater maximum effort (Andreacci et al., 2002). The encouragement cues were standardized in wording (e.g., “let’s go,” “keep going”) and remained the same across the days on which VE was applied during the testing procedure. No VE and no persons other than the researcher and the athletes were allowed to be present during the tests.

### Short Recovery and Stress Scale

To control for recovery-stress state and to exclude the potential confounding factor of “physical and/or mental fatigue,” the SRSS was included. Immediately prior to the start of the testing on every testing day, each athlete completed the SRSS. The SRSS (Kellmann et al., 2016; Kellmann and Kölling, 2019) consists of four recovery- and four stress-related items which are answered on a Likert-type rating scale ranging from 0 (*does not apply at all*) to 6 (*fully applies*). Recovery-related items are *Physical Performance Capability*, *Mental Performance Capability*, *Emotional Balance*, and *Overall Recovery*. Stress-related items are *Muscular Stress*, *Lack of Activation*, *Negative Emotional State*, and *Overall Stress*. Each item is supported by four adjectives (e.g., recovered, rested for *Physical Performance Capability*). Validity of the SRSS has been shown recently in field studies (Hitzschke et al., 2015) as well as laboratory settings (Pelka et al., 2017). Convergent validity is supported by correlations with the Acute Recovery and Stress Scale (ARSS) (Hitzschke et al., 2015; Kellmann et al., 2016). For the purpose of this study, only the scores of the items *Physical Performance Capability*, *Mental Performance Capability*, *Emotional Balance*, and *Overall Recovery* were analyzed.

SRSS analyses revealed that all of the recovery-related items across all of the four testing days did not differ within each item and between the athletes (*Physical Performance Capability*, 3.8 ± 0.7; *Mental Performance Capability*, 3.8 ± 0.7; *Emotional Balance*, 4.3 ± 0.7; and *Overall Recovery*, 3.5 ± 0.7; *p*-values ranging between 0.54 < *p* < 0.87).

### Statistical Analyses

Data are provided as means (*M*) and standard deviations (*SD*) and were initially tested for normal distribution (Shapiro–Wilks test) and homogeneity of variances (Levene test). The parameter for strength (1 RM squat in kg) and endurance [AMRAP, number of repetition (reps)] were analyzed by two separate repeated measures analysis of variance (rANOVA) between the VE and CON condition (condition effect). Two separate ANOVAS were calculated for each outcome parameter. Thereby, the best trial in strength testing and endurance testing during day 1 and day 2 for each condition was included into analyses. Main effects of condition were considered significant with *p* < 0.05. Effect sizes (Cohen’s *d*, trivial: *d* < 0.2, small: 0.2 ≤*d* < 0.5, moderate: 0.5 ≤*d* < 0.8, large *d* ≥ 0.8 (Cohen, 1992)) and the percentage changes of the performance values were additionally calculated.

The change scores between VE and CON were calculated with 90% confidence intervals (difference between the mean values for the VE and CON, divided by the average *SD* for both conditions; Glass, 1977).

Intraclass correlation coefficients (ICCs) were computed as a measure of relative reliability according to the formula ICC = 1 – (SEM^2^/SD^2^). The standard error of measurements (SEM) or typical error (TE, computed as the SD of the difference divided by the square root of 2) was calculated, along with the log-transformed coefficient of variation (CoV) together with 90% confidence intervals as measures of absolute reliability (Atkinson and Nevill, 1998; Hopkins, 2000). Reliability data were analyzed using a published spreadsheet from Hopkins in Microsoft^®^Excel (Hopkins, 2015).

Statistical analysis of the SRSS was computed for each of the four recovery-related items (*Physical Performance Capability*, *Mental Performance Capability*, *Emotional Balance*, and *Overall Recovery*) applying a repeated measures analyses of variance separately for each parameter and indicated as *M* and *SD* for each day and across all four testing days.

## Results

### Between-Day Reliability

Ranging between 0.87 and 0.99 with narrow confidence intervals, very good to excellent relative between-day reliability was observed for both strength and endurance parameters. Thereby, ICC values were slightly higher for strength testing (Table 2).

**Table 2:** Absolute and relative day-to-day reliability values for HIFT-specific strength (1 RM squat in kg) and endurance (repetitions, reps) tests in both test settings (CON vs. VE).

	Mean ±*SD*	*p*-value	CoV [90% CI]	TE [90% CI]	ICC [90% CI]
Squat (CON) day 1 (kg)	102.8 ± 33.1	0.99	3.5 [2.9; 5.0]	3.6 [2.9; 5.0]	0.99 [0.98; 0.99]
Squat (CON) day 2 (kg)	102.9 ± 31.1				
Reps (CON) day 1(reps)	171.2 ± 32.3	0.87	7.3 [5.7; 10.3]	12.8 [10.1; 17.8]	0.87 [0.73; 0.94]
Reps (CON) day 2 (reps)	176.4 ± 35.2				
Squat (VE) day 1 (kg)	103.3 ± 32.5	0.99	4.1 [3.2; 5.7]	3.6 [2.9; 5.0]	0.99 [0.98; 1.00]
Squat (VE) day 2 (kg)	105.5 ± 33.6				
Reps (VE) day 1 (reps)	169.8 ± 29.3	0.95	3.9 [3.1; 5.4]	7.5 [5.9; 10.4]	0.95 [0.89; 0.98]
Reps (VE) day 2 (reps)	181.4 ± 33.1				


*Notes: HIFT, high-intensity functional training; CoV, coefficient of variation; CI, confidence interval; TE, typical error; ICC, intraclass correlation coefficient; CON, non-encouragement condition; VE, verbal encouragement condition*.

Absolute reliability values for strength data computed as CoV ranged between 3.5 to 4.1% for CON and VE, respectively. CoV for endurance values were slightly lower in VE (3.9%) than in CON (7.3%) condition.

Interestingly, the CoV for reps with VE was slightly lower compared to CON.

### Strength Testing

Univariate comparison for mean squat strength revealed higher values for VE compared to CON (Table 3). Mean difference for squat strength revealed a slight difference of 2.0 kg (90% CI 0.8; 3.2) between VE and CON. Corroboratively, the Cohen’s *d* showed a trivial effect size (*d* = 0.06).

**Table 3:** Univariate comparison for maximal strength (squat) and endurance (reps) values for the control (CON) and encouragement (VE) condition.

		*Mean* ±*SD*	Mean difference [90% CI]	Cohen’s *d*	*p*-value
Strength (kg)	CON	104.7 ± 32.5	2.0 [0.8–3.2]	0.06	0.009^∗∗^
	VE	106.7 ± 33.1			
Endurance (reps)	CON	180.6 ± 35.0	1.2 [-4.4; 6.8]	0.03	0.71
	VE	181.8 ± 33.0			


*Notes: CI, confidence intervals; CON, non-encouragement condition; reps, repetitions; VE, verbal encouragement condition. ^∗∗^, significant effect of condition with *p* < 0.05*.

### Endurance Testing

The amount of repetitions during the endurance test showed only negligible differences between test conditions with slightly more repetitions during VE compared to CON (Table 3). The mean difference between VE and CON including 90% confidence intervals was 1.2 reps (90% CI -4.4; 6.8).

## Discussion

The aim of this study was to examine the occurrence and relevance of effects of VE during HIFT-specific strength and endurance testing in experienced HIFT athletes. We further intended to assess if potential effects would exceed between-day variability and whether VE affects reliability measures during those tests. HIFT-specific strength and endurance tests showed good to excellent between-trial reliability. Interestingly, VE led to higher reliability during endurance testing and 1 RM strength testing showed overall better absolute and relative reliability values. Based on these findings, adequate reliability measures during HIFT-specific strength and endurance testing enable a sufficient detection of cross-sectional or longitudinal changes of HIFT performance with and without VE.

Accompanied stress and recovery assessment by the SRSS indicated that the athletes were sufficiently recovered and that the perception of the individuals’ recovery and stress state did not potentially affect performance on testing days. This is an important prerequisite during strength and endurance testing, particularly regarding the risk of overtraining syndromes or when athletes are underperforming (Gustafsson et al., 2008).

### Verbal Encouragement and Strength

We only observed minimal differences in maximal squat weight changes between the two testing conditions. HIFT athletes performed merely 3–5 kg above their 1 RM in both CON and VE condition. Obmiñski and Mroczkowska (2015) obtained similar findings and assumed that maximal strength performance might not be sensitive enough to external verbal stimulation. This finding is also in line with Argus et al. (2011), who examined effects of VE on upper-body performance in elite rugby players and also found small effects of VE on strength performance. Thus, small improvement in strength for experienced athletes is difficult to detect under different encouragement conditions. In accordance with Obmiñski and Mroczkowska (2015), very small differences in strength metrics could also be explained by the fact that all participants in our study were very skilled athletes who may therefore be highly intrinsically motivated.

In contrast to our experienced sample of HIFT athletes, novice athletes may show greater differences in squat strength depending on technical and motivational deficiencies (Silva et al., 2013). In this regard, Argus et al. (2011) stated that psychological interventions may have greater potential to affect performance in untrained or novice athletes.

Another potential reason why VE may have not affected strength performance in our sample is given by Campenella et al. (2000). These authors reported difficulties in standardizing verbal stimulation in isokinetic strength testing of concentric quadriceps and hamstrings peak torque in males and females. Standardization of the verbal stimulus can be accomplished by using predetermined cues or by regulating the volume of the verbal commands using a sound-level meter, as it has been implemented previously (Kimura et al., 1999; Andreacci et al., 2002). Furthermore, other researchers demonstrated that an increase in volume of verbal commands (i.e., VE) is positively correlated with an increase in strength (Johansson et al., 1983). Further studies confirmed these positive effects in strength performance regarding encouragement volume and word choice in strength tests (McNair et al., 1996). Volume standardization was purposefully not implemented in our study. We commonly applied VE in an ecological valid way, similar to training and competition settings of HIFT. By using standardized tape recorded VE, appropriate individual feedback may be missed and produce conflicting results (Kimura et al., 1999).

Another explanation for an absent performance effect by VE application might be explained by muscular co-activation, evoked by verbal stimuli as presumed previously (Kellis and Baltzopoulos, 1996). In order to protect the knee, co-activation of the musculature around the knee joint increases stability and reduces joint displacement, which therefore can limit force production (Kellis and Baltzopoulos, 1996). Muscular co-activation was not measured in our study, although co-activation can be assumed to a certain degree while squatting.

Further explanations for the minimal increase in strength performance while VE in the present study might be the high intrinsic motivation and skill level of athletes in the present study. The proposed mechanisms which might occur during VE (e.g., startle mechanism, neuromuscular activation, and increased arousal level) did not contribute to practical relevant performance improvements due to high motivation and skill level of participants. Although the findings of Belkhiria et al. (2018) indicated that VE leads to increased activation and recruitment of motor units, including early recruitment and greater motor unit synchronization, leading to enhanced hand grip strength, this obviously does not translate to dynamic concentric and eccentric muscle contractions involving large muscle groups in trained athletes.

### Verbal Encouragement and Endurance

Minimal performance differences between CON and VE conditions were observed for endurance measures. Surprisingly, VE did not affect the endurance score for all athletes positively, as some athletes even scored lower when VE was applied. This finding might also be due to potential distracting effects (Brupbacher et al., 2014). Individual differences in response to VE could be explained by different personality traits, test anxiety, or intrinsic motivation in athletes of the investigated sample (Spray and Wang, 2001; Binboğa et al., 2013; Obmiñski and Mroczkowska, 2015). Effects of VE on endurance performance may be larger when investigating unexperienced athletes, as they have limited personal experience in performing maximally during all-out tests while more experienced athletes may not need external motivation to perform maximally (Moffatt et al., 1994; Andreacci et al., 2002; Karaba-Jakovljevic et al., 2007). This is in line with previous research (Karaba-Jakovljevic et al., 2007), which reported significant increases in all Wingate test parameters after the first test. This observation has been attributed to the fact that non-athletes have no personal experience about how maximal effort actually feels like and therefore performance on the first test day may be considered submaximal. Additionally, the chosen frequency for VE may have not been suited for endurance testing in the given HIFT setting. Encouragement frequency was chosen based on the findings of Andreacci et al. (2002), who reported VE every 20 s and every 60 s to elicit the greatest increase in maximal treadmill performance. Compared to treadmill tests, the technical requirements for the HIFT-specific endurance test were substantially higher. It can be assumed that VE every 60 s may have occurred too frequently and may have possibly interfered with the necessary focus required regarding technical execution of the exercises or that frequent VE may have elicited a saturation effect in some athletes (Andreacci et al., 2002). They further hypothesized that instructional encouragement cues could influence performance differently than motivational cues. The encouragement cues used in our study were not previously tested on instructional effects and therefore, no information can be provided in this regard.

Consequently, studies investigating the effect of VE on endurance performance using different tests and encouragement settings revealed inconsistent findings. On one hand, recent studies reported no significant effect of VE on performance during Wingate anaerobic test between female athletes and non-athletes (Bullinger et al., 2012). Additionally, duration of the Wingate anaerobic Test is only 30 s. Therefore, an encouragement frequency of 60 s is not possible and findings of longer lasting test efforts are difficult to compare to. On the other hand, the performance during longer test durations such as the 20 m shuttle run test (Dias Neto et al., 2015), using the same 60 s encouragement frequency as applied in our study, was positively influenced by VE. Findings of a study investigating the effects of VE every 60 s during the 6-min walking test in elderly patients suggested that other factors influencing performance aside from VE need to be considered (Marinho et al., 2014).

### Limitations

Several limitations in our study need to be addressed: (1) technical requirements in exercise execution may have interfered with external stimulation and therefore, the tests may have not been sensitive enough; (2) athletes may respond differently to VE but personality traits have not been recorded; (3) although athletes were experienced in training HIFT on a regular basis, we did not evaluate whether the investigated athletes participated in competitions regularly or if they were pursuing competitive goals in HIFT; and (4) a higher number of participants would have given more statistical power to the data interpretation and a contribution for generalizing the findings. However, participants were experienced and skilled in training HIFT and therefore represent the homogenous sample we were aiming at.

## Conclusion

The effects of VE on strength and endurance performance in experienced HIFT athletes seem to be negligible. HIFT-specific strength and endurance tests demonstrated very good to excellent between-day reliability, particularly for strength testing. Interestingly, endurance testing did not show a benefit from VE. Stress and recovery states were monitored and probably did not influence the athletes’ performance. Since individual athletes responded differently to the application of VE, it seems important to modify VE (e.g., frequency, wording, etc.) to the individual needs and preference of an athlete. Athlete’s individual needs and preferences for VE could be assessed by the coaches and subsequently an individualized program of VE could be applied by coaches and fellow athletes. To further elaborate the effect of VE, larger sample sizes and additional psychological factors (personal traits, affective valence, motivation, emotion regulation), which are known to possibly influence performance, should also be considered. With respect to the excellent reliability and variability data, cross-sectional and longitudinal changes of HIFT-specific strength and endurance performance can be most likely detected with and without VE. Thus, future interventional research on HIFT performance changes might be promising.

## Author Contributions

All authors listed have made a substantial, direct and intellectual contribution to the work, and approved it for publication.

## Conflict of Interest Statement

The authors declare that the research was conducted in the absence of any commercial or financial relationships that could be construed as a potential conflict of interest.

## Footnotes

1 https://www.eknz.ch/
